# Antimicrobial Resistance and Its Impact on Food Safety Determinants Along the Beef Value Chain in Sub-Saharan Africa—A Scoping Review

**DOI:** 10.3390/tropicalmed10030082

**Published:** 2025-03-20

**Authors:** Godfrey Musuka, Jairus Machakwa, Oscar Mano, Patrick Gad Iradukunda, Pierre Gashema, Enos Moyo, Amon Nsengimana, Shepherd Manhokwe, Tapiwa Dhliwayo, Tafadzwa Dzinamarira

**Affiliations:** 1 International Initiative for Impact Evaluation, Harare, Zimbabwe; 2 Division of Veterinary Services, Veterinary Public Health Branch, Causeway, Harare P.O. Box CY551, Zimbabwe; 3 Department of Public Health, University of the Western Cape, Bellville, Cape Town 7535, South Africa; 4 Rwanda Food and Drug Authority, Kigali P.O. Box 1948, Rwanda; gadpatrickiradukunda@gmail.com; 5 College of Medicine and Health Sciences, University of Rwanda, Kigali P.O. Box 4285, Rwanda; gashemapierre@gmail.com; 6 Department of Public Health Medicine, University of KwaZulu Natal, Durban 4041, South Africa; moyoenos@yahoo.co.uk; 7 Department of Pharmacy, University of Rwanda, Kigali P.O. Box 4285, Rwanda; 8 Department of Food Science and Nutrition, Midlands State University, Gweru P.O. Box 9055, Zimbabwe; 9 Department of Community Medicine, Midlands State University, Gweru P.O. Box 9055, Zimbabwe; 10 ICAP in Zimbabwe, Harare, Zimbabwe; 11 School of Health Systems and Public Health, University of Pretoria, Pretoria 0002, South Africa

**Keywords:** antimicrobial resistance, beef value chain, one health, antimicrobial use, food safety

## Abstract

Antimicrobial resistance (AMR) poses a significant threat to human, animal, and public health, particularly in Sub-Saharan Africa (SSA), where the beef sector is vital to food security and livelihoods. We conducted a scoping review to explore the determinants and impacts of AMR within the beef value chain in SSA, highlighting the challenges and progress in mitigating AMR risks in livestock production. This review identifies key factors contributing to AMR, including the overuse and misuse of antimicrobials, inadequate veterinary oversight, and weak regulatory frameworks. These practices are prevalent across various stages of the beef value chain, from farm to slaughterhouse, and are exacerbated by informal markets and insufficient infrastructure. Our findings also highlight the role of environmental factors, such as contamination of feed, water, and manure, in the spread of resistant pathogens. Additionally, gaps in AMR surveillance, education, and enforcement limit effective control measures in the region. While efforts to combat AMR have gained momentum in some countries, including the development of national action plans and surveillance systems, substantial challenges remain. These include poor adherence to antimicrobial guidelines, insufficient veterinary training, and the lack of integration between sectors. There is a need for targeted research to better understand antimicrobial misuse, socio-economic drivers, and the environmental pathways of AMR, as well as the need for stronger regulatory frameworks and cross-border cooperation. Addressing these challenges will be essential to safeguarding food safety, public health, and the sustainability of the beef industry in SSA.

## 1. Introduction

Antimicrobial resistance (AMR) represents a growing and complex global health threat that transcends human medicine, posing significant risks to animal health, food safety, and public health systems worldwide [1,2]. In Sub-Saharan Africa (SSA), livestock farming plays a pivotal role in the economic, cultural, and nutritional landscape. Consequently, the spread of AMR within the beef value chain is a critical concern [3]. The beef sector, integral to SSA’s agriculture-based economies, provides food security and livelihoods for millions of people. However, the widespread and often indiscriminate use of antimicrobials in beef production, combined with weak regulatory frameworks, inadequate veterinary services, and unsustainable farming practices, has created an environment conducive to the emergence of AMR in livestock and beef products [4,5,6].

In Sub-Saharan Africa, livestock farming plays a fundamental role in rural economies, contributing to food security, income generation, and employment. Cattle production, in particular, forms a substantial part of the agricultural sector, with beef being a major source of protein in many communities [7]. The beef value chain in SSA spans several stages, including production, slaughter, processing, distribution, and consumption [8,9]. At each of these stages, there are potential points of contamination with antimicrobial-resistant pathogens, which can undermine food safety and pose risks to human health. AMR threatens not only the sustainability of the beef industry in SSA but also the broader efforts to safeguard public health and nutrition in a region already grappling with numerous socio-economic challenges [3].

The use of antimicrobials in livestock production is intended to treat infections, prevent diseases, and promote growth. However, the unregulated use and overuse of antimicrobials are key factors driving the development of AMR [10]. In SSA, where veterinary services are often limited and regulatory oversight is weak, the inappropriate use of antimicrobials remains widespread. As a result, pathogens such as *Salmonella*, *E. coli*, *Campylobacter*, and *Staphylococcus* in cattle can develop resistance to commonly used antibiotics [5]. This resistance can then be transmitted through the beef value chain, from farm to table, posing significant risks to consumers and complicating the treatment of foodborne and other infectious illnesses.

AMR in the beef value chain in SSA is not only a problem for livestock health but also for food safety, with potentially far-reaching implications for human health. The overuse of antibiotics in cattle farming is particularly alarming because of the possibility of resistant pathogens contaminating beef products during production, slaughter, and processing. As antimicrobial resistance continues to spread, there is growing concern about the efficacy of treatment options for diseases caused by these resistant pathogens. In SSA, where access to healthcare and antibiotics is already limited, the emergence of resistant foodborne pathogens adds another layer of strain to already fragile healthcare systems.

The beef value chain in SSA is often characterized by informal markets, lack of standardization, and inadequate infrastructure, all of which contribute to the persistence and spread of AMR [11]. Smallholder farmers, who form the backbone of beef production in the region, frequently use antimicrobials as a preventive measure or inappropriately for growth promotion [12]. These practices often occur in the absence of oversight from veterinary authorities, who are sometimes unavailable, undertrained, or ill-equipped to enforce antimicrobial stewardship [13]. The lack of formalized supply chains and traceability mechanisms further complicates the identification and control of antimicrobial-resistant pathogens in beef products [14]. As a result, consumers may unknowingly be exposed to contaminated beef, increasing their risk of developing antimicrobial-resistant infections.

Understanding the factors contributing to AMR in the beef value chain in SSA is crucial for developing strategies to mitigate its impact. These determinants are multifaceted and span a variety of domains, including antimicrobial use, production practices, environmental factors, market conditions, and socioeconomic influences. This scoping review seeks to explore the determinants and impacts of AMR in the beef value chain across SSA, identifying the challenges and lessons learned in addressing this issue.

## 2. Methods

A scoping review methodology was employed to identify and map the existing literature on AMR and its impact on food safety determinants along the beef value chain in SSA. This review followed the guidelines proposed by Arksey and O’Malley [15], with adherence to the Preferred Reporting Items for Systematic Reviews and Meta-Analyses for Scoping Reviews (PRISMA-ScR) [16]. Additionally, the PAGER framework [17] was integrated into this analysis process to enhance the rigor and structure of this review by categorizing findings into five core areas: patterns, advances, gaps, evidence for practice, and research recommendations.

### 2.1. Search Strategy

A systematic search was conducted across multiple electronic databases, including PubMed, Scopus, Web of Science, and EMBASE, to capture a comprehensive set of studies on 3 January 2025. The following search terms were employed to identify relevant articles: (“Antimicrobial Resistance” OR “AMR” OR “Antibiotic Resistance” OR “Antimicrobial Use” OR “Antibiotic Use”) AND (“Beef Value Chain” OR “Livestock” OR “Cattle Farming”) AND (“Food Safety” OR “Contamination” OR “Public Health” OR “Risk”). No restrictions were placed on publication date or geographical location, though the language was limited to English. All relevant academic journal articles were considered. Citations were exported to EndNote (EndNote v20.2.1, Philadelphia, PA, USA) for deduplication, and two authors independently screened the titles and abstracts for relevance based on the review’s objectives.

### 2.2. Eligibility Criteria

The studies considered for inclusion met the following criteria shown in Table 1.

### 2.3. Data Extraction and Analysis Using the PAGER Framework

We employed the PAGER framework to understand the determinants of AMR along the beef value chain and the implications for food safety. The PAGER framework (Patterns, Advances, Gaps, Evidence for Practice, and Research Recommendations) provides a structured approach to analyzing and reporting the findings of scoping reviews, helping to organize the results in a clear and actionable way [17]. This framework allowed us to improve the transparency of our review, ensure a more systematic data synthesis, and guide the formulation of practical recommendations.

Two authors independently extracted data using a custom-designed form in Microsoft Excel. This form captured essential study characteristics (author, year, study design, geographic focus, sample size, study period) and specific data related to AMR determinants and food safety risks across the beef value chain. The data extraction tool is summarized in Table 2.

To ensure reliability and consistency, the extraction process was completed in duplicate, and discrepancies were resolved through discussion between the two reviewers. Following data extraction, a thematic analysis based on an inductive approach was used to identify key patterns and categorize the data according to conceptual similarities [18]. The PAGER framework was then applied to structure the analysis:

Patterns: Identifying recurring trends or common findings across studies regarding AMR and its impact on food safety in the beef value chain;

Advances: Document any recent innovations or progress in addressing AMR along the beef value chain, including new technologies or interventions;

Gaps: Highlighting areas where evidence is limited or lacking, particularly concerning the risk of AMR contamination at various stages of beef production;

Evidence for Practice: Identifying practical recommendations or solutions that can be applied to mitigate AMR risks in the beef value chain, such as improved regulatory oversight or alternative antimicrobial practices;

Research Recommendations: Suggest future research directions to address knowledge gaps, enhance AMR surveillance, and improve food safety along the beef value chain in SSA.

## 3. Results

### 3.1. Screening Results

Our database keywords search found 1424 potentially eligible articles. Following title screening, 87 articles were eligible for inclusion in abstract screening. Further, 23 duplicates were removed, leaving 64 articles to be included in the abstract screening. Following the abstract screening, 41 studies were excluded, leaving 23 articles for full-article screening. Five articles were excluded after full-article screening, and 18 articles were included in the data extraction [11,19,20,21,22,23,24,25,26,27,28,29,30,31,32,33,34,35].

### 3.2. Characteristics of Included Articles

All included studies reported evidence of antimicrobial resistance in the beef value chain in SSA. Of these, three were conducted in Tanzania [19,20,28], three from Kenya [23,29,34], two from Nigeria [21,24], and one each from Cameroon [22], Ethiopia [27], Malawi [25], South Africa [26], Uganda [11], and Zimbabwe [35]. Four were multi-country studies [30,31,32,33]. All included studies were published between 2019 and 2024. Study designs for included studies were as follows: 10 cross-sectional surveys [20,21,22,24,26,27,29,31,32,34], three qualitative [23,25,28], and four mixed methods [11,19,30,35]. One article was a report retrieved from a funding institution’s website [33].

### 3.3. Patterns: Determinants of AMR in the Beef Value Chain

The determinants of AMR in the beef value chain were categorized into four themes: (i) antimicrobial use and misuse, (ii) production practices and animal health management, (iii) market factors and informal beef trade, and (iv) environmental and waste management factors. The coded findings for patterns presented in the included studies are presented in Table 3.

AMR in SSA’s beef value chain is shaped by various patterns related to antimicrobial use, production practices, environmental factors, and socio-economic conditions. A notable pattern is the overuse and misuse of antimicrobials [19,20,21,22,23]. Studies in Tanzania have indicated that some farmers may not adhere to recommended withdrawal periods for veterinary drugs, as outlined by government regulations and drug manufacturers [19,20]. This non-compliance may stem from a lack of awareness about the potential public health risks and the perceived economic consequences of adhering to these periods. This practice can contribute to the development of AMR. For example, elevated levels of oxytetracycline residues have been detected in some ready-to-eat beef samples, exceeding the maximum residue limits established by the Food and Agriculture Organization (FAO) and the World Health Organization (WHO) [20]. As observed in many parts of SSA, antibiotics are often used as growth promoters or preventative treatments, even without disease [11,21,23,25].

In Nigeria, one study revealed that the use of antibiotics in farms is not always supervised or prescribed by veterinary professionals, especially in smallholder farming systems where access to veterinary services is limited [24]. This study identified three primary pathways for the spread of AMR from beef animals: consumption of contaminated meat, contact with infected animals and contaminated surfaces (fomites), and spread through contaminated manure and aerosols in the environment [24]. This study further revealed that inappropriate antimicrobial use, inadequate enforcement of relevant laws and regulations, low levels of education and expertise among relevant stakeholders, and poor farm management systems significantly drive antimicrobial misuse and the emergence of AMR [24].

In Malawi, the high dependence on antibiotics in small-scale intensive beef farming, driven by economic necessity and limited access to veterinary care, coupled with weak regulatory oversight and easy access to antibiotics, including critically important ones like colistin, were reported as important determinants of AMR [25]. This pattern of poor antimicrobial stewardship across various stages of beef production (from farm to slaughterhouse) contributes to the increased prevalence of AMR [19,21,22,24].

The structure of the beef market in SSA is another key determinant of AMR. In many countries, beef production and trade are characterized by informal, unregulated markets. In Uganda, a situational analysis reported that cattle may be sold through local markets, often with little or no oversight from regulatory bodies, and the traceability of beef products is limited [11]. Without standardization of slaughterhouse practices and meat processing, there is an increased risk of contamination with resistant pathogens during handling and preparation [24,26]. Moreover, the trade of beef products across borders within SSA may introduce additional complexities related to AMR. In some cases, imported beef from countries with different standards for antimicrobial use may introduce resistant pathogens into local beef markets. This cross-border trade in beef highlights the importance of regional cooperation and harmonization of regulations to control AMR effectively.

This review also revealed the significant role of the environment in the emergence and spread of AMR across various food production systems, including beef production. In the context of beef cattle, potential environmental sources of AMR include contaminated feed, direct or indirect contact with humans, contaminated water sources, airborne transmission through dust and aerosols, soil contaminated with manure or antimicrobial residues, wildlife, rodents, arthropods, and contaminated farm equipment. In Ethiopia, determinants of AMR in the beef value chain were reported as including educational status, job-related training, contamination risk perception, hygiene practices such as neatness of knives, source of contamination, and personal protective equipment use like wearing protective clothes [27]. Furthermore, proper handwashing techniques and safe money-handling practices were identified as crucial factors in reducing Salmonella contamination and subsequent AMR in butcher shops [27].

### 3.4. Advances: Efforts to Combat AMR

Efforts to combat AMR in Sub-Saharan Africa’s beef value chain have made notable strides through national action plans, farmer education, and strengthening regulatory frameworks [28,29,30]. While only a few countries have documented national AMU and AMR surveillance programs specifically for animals or the environment, there is an increasing number of countries in the region that have made significant strides by developing national antimicrobial plans as a first step toward building comprehensive surveillance systems for AMU and AMR across human, animal, and environmental sectors [28,30]. International organizations like WHO, FAO, and WOAH are providing crucial support to enhance the capacity for surveillance and monitoring of AMU and AMR in the beef value chain. One significant advance has been the use of enhanced AMR surveillance systems, which provide vital data on the occurrence and spread of resistant pathogens [33]. In Kenya, integrating vaccination strategies, especially for vaccine-preventable diseases, and improving access to veterinary services have played a crucial role in reducing unnecessary antimicrobial use and mitigating AMR risks in pastoralists in the country [34].

### 3.5. Gaps: The Challenges in Tackling AMR

A key gap in Africa’s efforts to tackle AMR in the beef value chain is the lack of infrastructure and institutional capacities, including insufficient laboratories, cold-chain systems, and facilities for effective surveillance and diagnostics. Additionally, a shortage of trained personnel, such as veterinarians and technicians, limits the implementation of AMR control measures and effective antimicrobial stewardship [28,30].

While there have been some positive efforts, significant gaps remain in addressing AMR effectively [28]. Frumence et al. reported significant data gaps in Tanzania’s AMR response, especially in weak reporting and feedback mechanisms, which hinder the tracking of resistance trends and the effectiveness of control measures [28]. In the beef value chain in SSA, such gaps prevent a clear understanding of AMR’s impact on food safety, as insufficient data on antibiotic use and resistant pathogens make it challenging to design effective interventions. This study also points to implementation gaps, including challenges with accountability, transparency, and sustainability of Tanzania’s National Action Plan (NAP) on AMR [28]. In the beef sector, these gaps can result in inadequate enforcement of antimicrobial use regulations, insufficient training for farmers and veterinarians, and poor monitoring of resistance in meat products, directly compromising food safety. Furthermore, the lack of sectoral integration, mainly the exclusion of the environmental sector from the NAP [28], highlights the fragmented nature of AMR efforts.

The gaps reported in Tanzania can be seen as representative of broader challenges across Sub-Saharan Africa. In many countries, weak governance and coordination can result in fragmented AMR policies that fail to adequately address the unique risks posed by the beef value chain. AMR in livestock production directly influences food safety, as beef-resistant pathogens can spread through improper handling, lack of hygienic practices, and inadequate veterinary oversight. The absence of environmental considerations in AMR frameworks further exacerbates these risks, as resistant bacteria can persist in the environment and contribute to the ongoing spread of AMR.

Researchers in a cross-sectional study conducted in Burkina Faso and Cameroon identified significant environmental gaps in tackling AMR in the beef value chain [32]. Reservoirs of AMR in livestock waste and treated wastewater, when spread on agricultural land, can contaminate the environment and contribute to the dissemination of resistant pathogens into the beef supply chain [32]. This creates persistent reservoirs of resistance in the soil, water, and feed, which can infect livestock, contaminate meat, and pose risks to human health. These environmental transmission pathways complicate efforts to control the spread of AMR, undermining mitigation strategies and exacerbating challenges in ensuring food safety across the beef value chain in Sub-Saharan Africa.

A shortage of infrastructure and institutional capacities hampers efforts to combat AMR effectively. Many countries lack well-equipped laboratories to conduct AMR testing, while limited cold-chain systems and inadequate facilities compromise surveillance and diagnostics [30,31,32]. Human resources are also lacking, as there is a shortage of adequately trained personnel, including veterinarians, laboratory technicians, and extension officers, severely limiting the implementation of AMR control measures [30,31].

A mixed methods study to investigate and determine the performance in addressing antimicrobial resistance in Kenya, Tanzania, Uganda, and Zambia revealed significant gaps in tackling AMR in SSA, including limited capacity in the animal, environmental, and agricultural sectors to conduct AMR surveillance [30]. There is also a lack of data on AMR across the region, with insufficient regional data sharing and uncoordinated research efforts that hinder the creation of a national database [30]. Additionally, poor adherence to recommended drug usage practices, such as incorrect dosages, wrong administration routes, and failure to follow withdrawal periods, exacerbates the spread of resistance [30]. Caudell et al. investigated knowledge, attitudes, and practices regarding antimicrobial use and AMR among pastoralist communities in Ghana, Kenya, Tanzania, Zambia, and Zimbabwe. Their study revealed key gaps in tackling AMR in Africa that include the misuse and abuse of antimicrobial drugs due to limited professional veterinary services and poorly regulated drug access [31].

### 3.6. Evidence for Practice: What We Know and What Works

Mounting evidence supports the need for a multifaceted approach to combating AMR in the beef value chain in SSA. Studies indicate that better antimicrobial use regulation and enforcement can significantly reduce resistance [31,33].

In Malawi, significant advances have been made in AMR surveillance, with the development of a fully functioning national system supported by the Fleming Fund, including the establishment of surveillance sites and the creation of the AMR National Coordinating Committee (AMRNCC) [33]. This has facilitated better policymaking and data-driven interventions, contributing to the country’s commitment to combating AMR across human, animal, and environmental health sectors.

In Kenya, the implementation of a One Health approach has been a key advancement, with strong national and county-level governance structures established, such as the National Antimicrobial Stewardship Interagency Committee (NASIC) and County Antimicrobial Stewardship Interagency Committees (CASICs) [33]. These efforts, alongside increased awareness and capacity-building initiatives, have helped improve surveillance, coordination, and compliance with antimicrobial regulations in both the agricultural and health sectors [33]. Caudell et al. highlight the need for bottom–up interventions tailored to local contexts, as well as improved engagement with animal health professionals to address AMR at the farm level effectively [31].

In Zimbabwe, the implementation of a vaccination program against theileriosis, a tick-borne disease, has helped reduce reliance on antibiotics for disease prevention in cattle [35]. This initiative has demonstrated that vaccinating livestock against certain diseases can reduce the need for antimicrobial interventions, thus minimizing the risk of AMR development [35].

### 3.7. Research Recommendations: The Path Forward

Based on the findings of the included studies, there are several key research recommendations for addressing AMR in the beef value chain across SSA. First, there is a need for comprehensive studies on antimicrobial use and misuse, particularly in smallholder farming systems, to understand the underlying factors driving inappropriate practices, such as improper dosage, wrong administration routes, and failure to observe withdrawal periods [21,24,25]. Caudell et al. recommend further research to understand farmers’ knowledge, attitudes, and practices regarding antimicrobial use, particularly in pastoralist communities [31]. In Western Kenya, researchers recommended further research to identify the sources and pathways of AMR transmission in slaughterhouses, as well as to determine critical intervention points and surveillance strategies along the food chain [29]. Research should also explore the socio-economic drivers of antimicrobial misuse, especially in areas with limited access to veterinary services and regulatory oversight. Additionally, studies focusing on the role of environmental factors, such as the contamination of soil, water, and feed with antimicrobial residues, would provide valuable insights into the transmission pathways of AMR within the beef supply chain [32].

Furthermore, there is a significant need for research to strengthen the capacity of institutions involved in AMR surveillance and control. This includes studies on improving laboratory infrastructure, cold-chain systems, and training for veterinary professionals, laboratory technicians, and extension officers, which are essential for effective AMR diagnostics and management [30,31]. Future research should also focus on integrating environmental considerations into AMR frameworks, particularly with agricultural and wastewater management practices. Finally, cross-border studies investigating the impact of informal beef trade and the harmonization of regulatory standards across SSA would be valuable in addressing the regional complexities of AMR in the beef value chain [11].

## 4. Discussion

This study explored the determinants and impacts of AMR in the beef value chain across SSA, identifying four key themes: antimicrobial use and misuse, production practices and animal health management, market factors and informal beef trade, and environmental and waste management factors. The findings reveal that AMR in SSA is driven by the overuse of antibiotics for growth promotion and disease prevention, weak regulatory frameworks, informal markets, and environmental contamination. Despite some progress in national action plans and surveillance systems, significant infrastructure, governance, and data availability gaps hinder effective AMR control. This study also highlights the importance of a One Health approach to address the interconnected drivers of AMR across human, animal, and environmental health sectors.

The findings underscore the critical role of socio-economic and environmental factors in shaping AMR in SSA’s beef value chain. In Brazil, Canada, the United States of America, Argentina, the European Union, and Australia, some of the world’s leading beef producers, antimicrobials are also widely used to treat a wide variety of conditions [36].

The widespread misuse of antimicrobials, particularly in smallholder farming systems, reflects a lack of awareness, economic pressures, and limited access to veterinary services. This aligns with global evidence that highlights the link between unregulated antibiotic use and the emergence of resistant pathogens [37]. The detection of antibiotic residues in beef products exceeding recommended limits further emphasizes the public health risks associated with AMR, particularly in regions with fragile healthcare systems [38]. According to research by Kumar et al., 2024, an increase in bacteria that are resistant to antibiotics has contributed to an increase in the number of illnesses that are caused by consuming contaminated food [39]. Furthermore, antibiotic residues in food have been linked to a range of adverse effects, including allergic reactions, hepatotoxicity, mutagenicity, carcinogenicity, toxic effects, nephropathy, and the development of antibacterial resistance [39,40]. Consequently, antibiotic-resistant bacteria and infectious illnesses precipitate heightened risks of treatment failure, prolonged illness duration, increased healthcare costs, and elevated mortality rates and impose a significant burden on public health systems [40]. Factors such as treatment delays or failures, limited access to effective antibiotics, persistence of drug-resistant strains during treatment, and the co-existence and increased virulence of resistance genes collectively exacerbate this issue [41].

This study’s findings suggest that current practices in antimicrobial use, coupled with deficiencies in market regulation and environmental management, create significant risks that compromise food safety in SSA’s beef value chain. These risks are multifaceted, stemming from the significant challenges identified in this study. Contamination of beef products with drug-resistant pathogens such as *Salmonella*, *E. coli*, and *Campylobacter* directly threatens food safety, as consumers may ingest these pathogens through improperly handled or undercooked meat. These pathogens are known to be causative agents for various illnesses, and the WHO has classified some as presenting a significant threat to humans [42].

The detection of antibiotic residues, such as oxytetracycline, in beef samples exceeding maximum residue limits further exacerbates the risk, as prolonged exposure to low levels of antibiotics can contribute to the development of resistant infections in humans [43]. The risk of acquiring antibiotic-resistant genes (ARGs) from eating beef or contaminated meat is a major concern [44]. The informal nature of beef production and trade in SSA also contributes to food safety risks. This study notes that informal markets often operate without regulatory oversight, leading to poor hygiene practices, inadequate slaughterhouse conditions, and limited traceability of beef products. These factors increase the likelihood of contamination with resistant pathogens during handling, processing, and distribution [45]. Unregulated markets and cross-border trade further create opportunities for spreading resistant pathogens through contaminated meat.

This study’s emphasis on the One Health approach is particularly significant, as it underscores the need for integrated strategies that address the interconnected drivers of AMR. While previous studies have focused primarily on human and animal health, this study highlights the critical role of environmental factors, such as contaminated water and soil, in perpetuating AMR [46]. Environmental factors tie into the whole beef value chain as they start with contamination on the farm and go as far as meat contamination during and after slaughtering. This is because most slaughterhouses in SSA are informal and ill-equipped to adhere to proper hygiene standards during the process of slaughtering and distribution [29]. On the farm, antibiotic-resistant bacteria and ARGs are spread further when cattle manure is utilized as soil fertilizer [47]. These findings have important implications for policy and practice, as they call for greater attention to environmental management in AMR control efforts.

The gaps identified in this study, such as weak governance, limited infrastructure, and insufficient data, are consistent with broader challenges in addressing AMR in LMICs. Furthermore, this study also highlights the potential for regional collaboration and harmonization of regulations to address the cross-border spread of AMR. This requires thinking beyond National Action Plans and bringing international harmonization of regulations into the conversation.

In response to the growing concerns over AMR, exploring alternatives to antibiotic use in beef production is imperative. Natural growth promoters (NGPs), such as probiotics, prebiotics, organic acids, phytogenics, and tannins, have emerged as viable substitutes. These NGPs offer benefits, including enhanced gut health, improved growth performance, and strengthened immune responses in livestock without contributing to antibiotic resistance [42]. Additionally, bacteriophage therapy presents a promising alternative; bacteriophages are viruses that specifically target and eliminate bacterial pathogens, offering a targeted approach to infection control [43]. Implementing such alternatives requires a comprehensive understanding of their efficacy, safety, and integration into existing production systems. Moreover, preventive measures like improving animal husbandry practices, enhancing biosecurity, and ensuring better living conditions can reduce the necessity for antibiotic use, thereby mitigating AMR risks.

While this study provides valuable insights into the determinants and impacts of AMR in SSA’s beef value chain, it has some limitations. The reliance on existing studies and secondary data may limit the generalizability of the findings, as data availability and quality vary across countries. This study focuses primarily on the beef value chain, which may not fully capture the dynamics of AMR in other livestock sectors, such as poultry and piggeries. The emphasis on environmental determinants of AMR is also limited by the lack of data on environmental contamination and its impact on human and animal health, which can be an area of focus for subsequent studies.

From the findings of this paper, we recommend first that comprehensive studies on antimicrobial use and misuse, particularly in smallholder farming systems, be conducted to understand the underlying factors driving inappropriate practices, such as improper dosage, wrong administration routes, and failure to observe withdrawal periods [21,24,25]. Caudell et al. recommend further research to understand farmers’ knowledge, attitudes, and practices regarding antimicrobial use, particularly in pastoralist communities [31]. We further recommend strengthening regulatory frameworks by enforcing more stringent regulations on antimicrobial use, including adherence to withdrawal periods and maximum residue limits, to reduce the misuse of antibiotics. Farmer and consumer knowledge of AMR must be enhanced through the implementation of education programs to raise awareness about the risks of AMR and promote best practices in livestock management. This will also sensitize the consumer regarding the risks associated with consuming meat products from sources of questionable repute. Improving access to veterinary care and training for farmers and veterinarians will support antimicrobial stewardship, and integrating human, animal, and environmental health strategies to address the interconnected drivers of AMR will provide valuable data for policy-makers. Harmonizing regulations and promoting data sharing across countries must be encouraged to address the cross-border spread of AMR in the region.

This study highlights AMR’s complex and multifaceted nature in SSA’s beef value chain, emphasizing the need for a coordinated, multi-sectoral approach to address this growing public health threat. By integrating regulatory, educational, and environmental strategies, SSA can mitigate the impact of AMR on food safety, public health, and the sustainability of its beef industry. Future research and policy efforts should focus on filling data gaps, strengthening governance, and fostering regional collaboration to combat AMR in the region effectively.

## 5. Conclusions

This study demonstrates that the overuse and misuse of antimicrobials, suboptimal production practices, informal market structures, and poor environmental management significantly contribute to AMR in SSA’s beef value chain. These conditions facilitate the emergence and spread of resistant pathogens while compromising food safety and public health. The findings underscore the urgent need for an integrated One Health approach that enhances regulatory oversight and antimicrobial stewardship. Although progress has been made in surveillance and policy, considerable gaps in infrastructure, capacity, and market formalization remain. Addressing these challenges is essential for ensuring the sustainability of the beef industry and the safety of the region’s food supply.

## Figures and Tables

**Table 1:** Inclusion and exclusion criteria.

Inclusion Criteria	Exclusion Criteria
Studies focused on antimicrobial resistance in the beef value chain, specifically in SSA.	Studies focused solely on AMR in human or environmental settings without reference to the beef value chain.
Research assessing the impact of AMR on food safety risks, including contamination risks at various stages of the beef value chain (e.g., production, slaughter, processing, and distribution).	Research on the molecular aspects of AMR, such as genomics, without an explicit focus on food safety risks.
Articles addressing factors such as antimicrobial use, production practices, and regulatory frameworks related to AMR and food safety.	Systematic reviews, meta-analyses, and secondary data sources were excluded from this review.
Studies published in English.	

**Table 2:** Data extraction tool.

Category	Description
Study Characteristics	Basic citation details, study design, geographical location, and study design.
Determinants of AMR *	Factors influencing AMR include antimicrobial use (types, administration methods, frequency), production practices (hygiene, veterinary care, biosecurity), market dynamics, environmental conditions, and socio-economic factors.
Food Safety Impacts	Assessing the contamination risks and public health implications linked to AMR.
Regulatory and Policy Frameworks	Outlining relevant regulations, enforcement levels, and international guidelines.
Technological and Innovative Interventions	Innovations or technologies used to mitigate AMR in the beef value chain.
Findings, Recommendations, and Study Limitations:	Summarizing the main findings, recommendations for policy or practice, and study limitations.

* AMR—antimicrobial resistance.

**Table 3:** Determinants of AMR in the beef value chain.

First Author, Year	Country	Study Design	Determinants of AMR in the Beef Value Chain
Mdegela, 2021 [19]	Tanzania	Mixed-methods	Farm practices and the availability of antimicrobials in livestock farming
Bilashoboka, 2019 [20]	Tanzania	Cross-sectional	Farmer non-compliance with veterinary drug withdrawal periods, driven by a lack of awareness and perceived economic losses, contributes to antimicrobial resistance, as evidenced by elevated oxytetracycline residues in some ready-to-eat beef.
Alhaji, 2023 [21]	Nigeria	Cross-sectional	Antimicrobial use and misuse
Mouiche, 2024 [22]	Cameroon	Cross-sectional	The overuse of antibiotics, disregard for the time required for withdrawal following antibiotic administration, and disregard for veterinarian advice
Kariuki, 2023 [23]	Kenya	Qualitative	Antimicrobial use and misuse
Alhaji, 2022 [24]	Nigeria	Cross-sectional	Production practices and animal health management
Mankhomwa, 2022 [25]	Malawi	Qualitative	Production practices and animal health management
Mubiru, 2023 [11]	Uganda	Mixed-methods	Farm practices and the availability of antimicrobials in livestock farming; market factors and informal beef trade
Jaja, 2020 [26]	South Africa	Cross-sectional	Market factors and informal beef trade
Geresu, 2021 [27]	Ethiopia	Cross-sectional	Production practices and animal health management

## Data Availability

No new data were created or analyzed in this study. Data sharing is not applicable to this article.
